# Human Activity Profile Questionnaire: functional assessment and screening for systolic dysfunction in patients with Chagas cardiomyopathy

**DOI:** 10.1590/0037-8682-0251-2024

**Published:** 2025-08-08

**Authors:** Lucas Frois Fernandes Oliveira, Matheus Ribeiro Ávila, Whesley Tanor Silva, Sueli Ferreira da Fonseca, Endi Lanza Galvão, Cheyenne Alves Fonseca, Vanessa Amaral Mendonça, Ana Cristina Rodrigues Lacerda, Sabrina Pinheiro Tsopanoglou, Sanny Cristina de Castro Faria, Daniel Menezes de Souza, Mauro Felippe Felix Mediano, Marcus Alessandro de Alcantara, Pedro Henrique Scheidt Figueiredo, Manoel Otávio da Costa Rocha, Henrique Silveira Costa

**Affiliations:** 1 Universidade Federal dos Vales do Jequitinhonha e Mucuri, Programa de Pós-Graduação em Reabilitação e Desempenho Funcional, Diamantina, MG, Brasil.; 2 Universidade Federal de Minas Gerais, Programa de Pós-Graduação em Ciências da Saúde: Infectologia e Medicina Tropical, Belo Horizonte, MG, Brasil.; 3 Instituto Nacional de Infectologia Evandro Chagas, Programa de Pós-Graduação em Pesquisa Clínica em Doenças Infecciosas, Rio de Janeiro, RJ, Brasil.; 4 Universidade Federal dos Vales do Jequitinhonha e Mucuri, Faculdade de Medicina, Diamantina, MG, Brasil.; 5 Universidade Federal dos Vales do Jequitinhonha e Mucuri, Departamento de Fisioterapia, Diamantina, MG, Brasil.

**Keywords:** Chagas disease, Chagas cardiomyopathy, Exercise, Activities of daily living, Human activities

## Abstract

**Background:**

Patients with chronic Chagas cardiomyopathy (CCC) may present with fatigue and dyspnea, which contribute to functional impairment. However, simple and inexpensive methods for evaluation of functional impairment and identification of left ventricular (LV) systolic dysfunction in patients with CCC are lacking. The Human Activity Profile (HAP) has potential in functional evaluation of patients with CCC. This study was conducted to analyze the association between HAP, functional parameters, and LV systolic dysfunction in patients with CCC, and to demonstrate the accuracy of HAP in identifying LV systolic dysfunction in patients with CCC.

**Methods:**

One hundred and twenty-six patients with CCC (NYHA I-III, 18.9% with LV systolic dysfunction) were evaluated using echocardiography, the 60-second sit-to-stand test (STS60, for lower limb strength and endurance), and the HAP questionnaire. In addition, the gait speed and handgrip strength of each patient was measured.

**Results:**

HAP score was correlated with gait speed (r=-0.206; p=0.023), STS60 score (r=0.199, p=0.030), and handgrip strength (r=0.315, p<0.01). Binary logistic regression showed that HAP score was the only functional variable associated with LV systolic dysfunction. Patients with LV systolic dysfunction (n=24) had lower HAP scores than those without LV systolic dysfunction (n=102) (p <0.01). The area under the ROC curve indicated that HAP score had an acceptable discriminatory ability to identify LV systolic dysfunction in patients with CCC (AUC=0.713). The optimal cut-off HAP score for identifying these patients was <56 points.

**Conclusion:**

HAP score is associated with LV systolic dysfunction in patients with CCC.

## INTRODUCTION

Chagas disease is an infectious disease caused by the protozoan *Trypanosoma cruzi* ^1^. According to the Pan American Health Organization^2^, approximately 6-8 million people in Latin America are infected with *T. cruzi*, and more than 10 thousand people die each year due to clinical complications. Among the infected patients, 30%-40% have the cardiac form of the disease, which is known as chronic Chagas cardiomyopathy (CCC)^3^, the most severe clinical manifestation of the disease.

Patients with CCC may present with a wide spectrum of clinical manifestations, ranging from electrical conduction disorders to cardiac chamber dilation with compromised ventricular systolic function^4^. Several systematic reviews^5^ ^-^ ^7^ conducted to identify the predictors of mortality in patients with CCC have established that left ventricular (LV) systolic dysfunction is an indicator of disease severity and an important marker of worse prognosis.

Fatigue and dyspnea are common clinical findings that can affect functional capacity and the ability to perform daily activities. A systematic review and meta-analysis^8^ demonstrated that functional impairment occurs during the early stages of heart disease. Furthermore, patients with heart disease typically report symptoms related to difficulties performing physical activities^9^ ^,^ ^10^, highlighting the importance of functional assessments for patients with CCC. 

Maximal tests, such as the cardiopulmonary exercise test, are indicated for functional assessment of patients. However, they are expensive and not available as routine clinical tests for patients in endemic areas. The potential value of field tests for functional assessment of patients has been demonstrated^11^. However, these tests require physical space and patient understanding. Furthermore, their associations with disease progression may be limited^12^.

A recent study^13^ demonstrated an association between the Human Activity Profile (HAP) and functional capacity in patients with CCC, indicating the ability of the HAP questionnaire to identify patients with functional impairment. The HAP questionnaire^14^ is a simple and inexpensive tool used for clinical and functional assessment of patients with CCC. The questionnaire, which was first described by Fix and Daughton^14^, comprises 94 items and is widely used for the assessment of patients with chronic diseases^15^, including stroke^16^ and heart failure^17^. It is easy to complete, can estimate physical fitness and energy spent on daily activities^18^ ^,^ ^19^, and has psychometric properties consistent with populations already studied^15^. The HAP questionnaire is considered a valid tool for evaluating functional performance, with items and domains related to self-care, work, social activities, and energy expenditure in different muscle groups^15^. However, to the best of our knowledge, the association between HAP score and functional parameters, such as gait speed, sit-to-stand test score, and hand grip strength, and its value in identifying patients with CCC and LV systolic dysfunction, have not yet been investigated.

In areas where Chagas disease is endemic, low-cost instruments with good applicability are important for risk stratification, screening, and assessment of disease severity. Therefore, the objective of this study was to analyze the association between HAP score, functional parameters, and LV systolic dysfunction in patients with CCC, and to verify the accuracy of HAP score in identifying LV systolic dysfunction in these patients. 

## METHODS

### Study design and ethical considerations

This was a cross-sectional study of patients clinically diagnosed with CCC. This study was conducted at the Cardiovascular Physiotherapy Laboratory at Universidade Federal dos Vales do Jequitinhonha e Mucuri (UFVJM) in Minas Gerais, Brazil, a region where Chagas disease is endemic. This study was conducted according to the ethical principles of the Declaration of Helsinki and approved by the Ethics Committee of UFVJM (CAAE 61176022.4.0000.5108). All included patients signed informed consent forms. The recommendations of the STARD 2015 Guidelines For Reporting Diagnostic Accuracy Studies were followed^20^ as appropriate.

### Study population

The inclusion criteria for this study were as follows: (1) a serological diagnosis of Chagas disease based on at least two positive serological tests for antibodies against *T. cruzi*, and (2) clinical, electrocardiographic, or echocardiographic findings compatible with CCC^21^. The exclusion criteria were the presence of systemic or cardiac disease caused other factors, inability to perform functional tests, or lack of understanding of the administered questionnaires. 

Sample size calculations were performed using the methods proposed by Zhou et al. for diagnosis studies^22^. Considering an area under the receiver operating characteristic (ROC) curve of at least 0.7, and a 1:4 ratio of patients with LV systolic dysfunction to patients without LV systolic dysfunction, the calculation indicated that a minimum sample size of 67 patients was needed. 

### Measurements

The selected patients underwent clinical evaluation following a structured protocol of anamnesis, completing the HAP questionnaire, echocardiography, measurement of gait speed, 60-second sit-to-stand test (STS60), and handgrip dynamometry. LV systolic dysfunction was defined as a left ventricular ejection fraction (LVEF) below 52% for mean and 54% for women^23^. All evaluations were performed on the same day by different researchers who were blinded to the results of the other assessments.

### Echocardiography

Echocardiography was performed according to guidelines of the American Society of Echocardiography^23^. The images were acquired using a Philips HDI 5000-ATL echo machine (Bothell, Washington, USA), and LVEF and left ventricular end-diastolic diameter (LVDd) were recorded. LVEF was obtained used the modified Simpson’s rule. Early diastolic velocity (e’) at the medial border of the mitral annulus was measured and the ratio between peak mitral E and e’ (E/e’ ratio) was calculated.

### Human Activity Profile

The HAP is a questionnaire with 94 items that evaluates a patient’s level of physical activity based on energy consumption during daily activities^14^ ^,^ ^15^. Patients’ responses are rated according to the adjusted activity score as “inactive” (less than 53 points), “moderately active” (53-74) points, and “active” (higher than 74 points)^15^. The target variable was the numerical score.

### Gait speed

Gait speed is considered a functional vital sign^24^ and a predictor of mortality in older adults^25^. Gait speed test was conducted in the present study in accordance with the protocols reported by Karpman et al.^26^. The patients were instructed to walk at their usual speed in a corridor for a distance of four meters. The target variable was the time spent covering the four-meter distance.

### 60-second sit-to-stand test

The STS60 test assesses muscular strength and endurance. In the present study, the test was conducted according to the protocols recommended by Strassmann et al.^27^. Each patient was instructed to sit and stand up from a chair, with arms crossed in front of the chest, for 1 minute. A pretest was performed to ensure safety. The target variable was the number of repetitions achieved in one minute. 

### Handgrip strength

Handgrip strength was measured using the hydraulic dynamometer JAMAR^®^ (Lafayette Instrument Company, Indiana, IN, USA), following the protocols proposed by Delinocente et al*.* ^28^. Each patient performed the test in a sitting position, with the elbow at 90 ° flexion, and was instructed to hold the dynamometer with the dominant hand as tightly as possible. The average of three measurements was calculated. The variable of interest was grip strength in kilograms. The highest of the three measurements was used in the analysis. 

### Statistical analysis

Statistical analysis was performed using Statistical Package for Social Sciences (SPSS) version 23.0 (IBM Corp., Armonk, NY, USA). The Kolmogorov-Smirnov test was used to verify data normality. Continuous variables are expressed as means and standard deviations or medians and interquartile ranges, whereas categorical variables are expressed as absolute numbers and percentages. 

The association between HAP score and functional variables was verified using the Pearson or Spearman correlation test. The t-test or Mann-Whitney U test was performed to determine the difference in means between groups (LV systolic dysfunction and preserved cardiac function groups). Binary logistic regression was performed to verify the association between LV systolic dysfunction (dependent variable) and functional parameters adjusted for sex and age. A significance level of 5% was adopted for all analyses. The assumptions of the binary logistic regression verified the linear relationship between the independent variables and log(odds), the absence of multicollinearity (based on the variance inflation factor), the absence of extreme factors, and variance modeled by the logistic function.

A ROC curve was constructed to verify the accuracy of HAP score in identifying LV systolic dysfunction. An area under the curve (AUC) greater than 0.7 was classified as adequate^29^. The Youden index was used to define the best sensitivity and specificity, and thus, the optimal cutoff point. Other factors with high sensitivity or specificity are also reported in this study. Sensitivity, specificity, positive predictive value, negative predictive value, and their respective 95% confidence intervals were obtained using MedCalc software version 13.1.2.0 (MedCalc Software, Ostend, Belgium).

## RESULTS

A total of 166 patients were evaluated for inclusion into this study. Of these, 40 patients had apparent heart disease and were excluded. Therefore, 126 patients were included in this study. Of the 126 patients, 24 (18.9%) had LV systolic dysfunction. The baseline characteristics of the patients are shown in Table 1.

**TABLE 1:** Baseline characteristics of the patients (n=126).

Variables	Value
Age (years)	63.1 [8.7]
Female sex (%)	79 (62.2)
Disease stage, n (%)	
B1	102 (80.9)
B2	14 (11.1)
C	10 (08.0)
NYHA functional classification, n (%)	
I	78 (61.4)
II	36 (28.3)
III	12 (9.4)
HAP (score)	61.2 [11.5]
Active, n (%)	26 (20.5)
Moderately active, n (%)	83 (65.4)
Inactive, n (%)	18 (14.2)
Functional variables	
Gait speed (seconds)	3.8 [0.9]
STS60 (repetition)	20.1 [4.6]
Handgrip dynamometry (kg)	33.9 [7.6]
Echocardiography	
LVEF (%)	63.0 (24 - 80)
LV systolic dysfunction, n (%)	24 (18.9)
LVDd (mm)	49.2 [6.6]
E/e’ ratio	9.0 (4.1 - 24.5)

Data are presented as the mean and SD [standard deviation], median (interquartile range), or absolute number (percentage). **NYHA:** New York Heart Association functional classification; **HAP:** Human Activity Profile questionnaire; **STS60:** 60-second sit-to-stand test; **LVEF:** left ventricular ejection fraction; **LVDd:** left ventricular end-diastolic diameter; **E/e’ ratio:** ratio of the early diastolic transmitral flow velocity to early diastolic mitral annular velocity.

Analysis of the functional variables indicated that HAP score was weakly correlated with gait speed (r=-0.206; p=0.023), STS60 score (r=0.199, p=0.030), and handgrip strength (r=0.315, p<0.01).

The binary logistic regression analysis indicated that HAP score was the only functional variable associated with LV systolic dysfunction, even after adjustment for age and sex (Table 2). Stratification of the patients according to cardiac function revealed that patients with LV systolic dysfunction had lower HAP scores (p-value=0.01) than those with preserved cardiac function (Table 3). Furthermore, patients with LV systolic dysfunction had a lower LVEF, higher LVDd, and higher E/e' ratio than those with preserved cardiac function (all p<0.01).

**TABLE 2:** Logistic regression analysis of the association between LV systolic dysfunction and functional variables adjusted for age and sex.

Variable	OR	95% CI	p-value
HAP	0.920	0.867-0.977	**0.01**
Gait speed	0.925	0.514-1.665	0.79
STS60	1.015	0.902-1.143	0.80
Handgrip dynamometry	0.964	0.884-1.050	0.39

Abbreviations: **OR:** Odds ratio; **95% CI:** 95% confidence interval; **HAP:** Human Activity Profile; **STS60:** 60-second sit-to-stand test.

**TABLE 3:** Functional and echocardiographic differences between patients with LV systolic dysfunction and patients with preserved cardiac function.

Variable	Preserved cardiac function (n=102)	Systolic dysfunction (n=24)	p value
Female sex, n (%)	65 (63.7)	13 (54.2)	0.39
Age (years)	62.8 [8.8]	65.7 [6.8]	0.11
**NYHA class**			
I	69 (67.6)	9 (37.5)	**<0.01**
II	30 (29.4)	6 (25.0)	
III	03 (2.9)	9 (37.5)	
**Functional variables**			
HAP (score)	64.0 [9.6]	56.3 [8.5]	**0.01**
Gait speed (seconds)	3.7 [0.8]	3.8 [0.9]	0.88
STS60 (repetition)	20.2 [4.5]	20.7 [4.7]	0.90
Handgrip dynamometry (kg)	34.5 [7.2]	34.1 [9.5]	0.21
**Echocardiography**			
LVEF (%)	65.6 [6.3]	40.2 [7.2]	**<0.01**
LVDd (mm)	47.0 [4.3]	59.2 [7.0]	**<0.01**
E/e’ ratio	8.8 (4.1 - 17.0)	11.3 (6.7 - 24.5)	**<0.01**

Data are presented as mean and SD [standard deviation]. Abbreviations: **NYHA:** New York Heart Association; **HAP:** Human Activity Profile questionnaire; **STS60:** 60 seconds sit-to-stand test; **LVEF:** left ventricular ejection fraction; **LVDd:** left ventricular end-diastolic diameter; **E/e’ ratio:** ratio of the early diastolic transmitral flow velocity to early diastolic mitral annular velocity. *Values in bold indicate statistical difference between groups.

**FIGURE 1:**
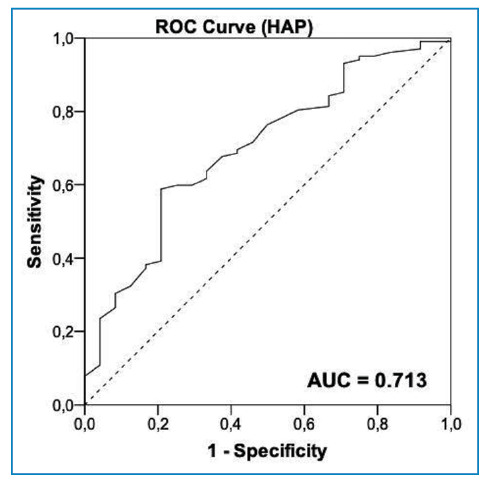
Area under the receiver operating characteristic curve demonstrating the accuracy of HAP score in identifying LV systolic dysfunction in patients with CCC.

The AUCs demonstrated that the HAP score had an acceptable discriminatory ability to identify LV systolic dysfunction in patients with CCC (AUC=0.713) (Figure 1). The optimal cut-off HAP score was <56 points. The sensitivity, specificity, positive predictive value, and negative predictive values obtained in the analyses are listed in Table 4.

**TABLE 4:** Cutoff, sensitivity, specificity, positive predictive value, and negative predictive values of HAP score for the identification of systolic dysfunction in patients with CCC.

Cutoff	Sensitivity % (95% CI)	Specificity % (95% CI)	PPV % (95% CI)	NPV % (95% CI)
37	33.33 (15.63-55.32)	81.52 (72.07-88.85)	32.00 (18.79-48.91)	82.42 (77.66-86.34)
56	79.17 (57.85-92.87)	42.16 (32.44-52.34)	24.36 (19.83-29.54)	89.58 (79.24-95.09)
70	95.83 (78.88-99.89)	11.96 (6.12-20.39)	22.12 (20.24-24.11)	91.67 (59.88-98.78)

Abbreviations: **CI:** confidence interval; **PPV:** positive predictive value; **NPV:** negative predictive value.

## DISCUSSION

To the best of our knowledge, this is the first study conducted to evaluate the use of the HAP questionnaire for identifying LV systolic dysfunction in patients with CCC. The results of this study validated the association between HAP score and functional variables that are easily measured in clinical practice. Specifically, the results of the present study demonstrated that HAP score is correlated with gait speed, lower limb muscle strength, and hand grip strength, and that HAP score shows acceptable accuracy in identifying LV systolic dysfunction in patients with CCC. These results demonstrate the potential value of this low-cost instrument for screening and risk stratification of patients with CCC.

The HAP questionnaire is a simple tool used for assessing functional performance in different populations and comprises 94 items related to the activities of daily living. A recent study^12^ showed that HAP score is strongly correlated with functional capacity in patients with CCC and can identify individuals with low peak oxygen uptake. This association can be explained by the fact that HAP score is based on estimated energy consumption during activities, which is closely related to exercise capacity^30^. However, other functional variables such as gait speed and peripheral muscle strength are also important for the performance of daily activities. All the variables analyzed in the present study were significantly correlated with HAP score; however, the correlations were weak. We hypothesized that a patient’s functional performance is a multifactorial construct, and that other variables may affect the ability to perform daily activities. Notably, a previous study^31^ demonstrated an association between HAP score and the presence of depressive symptoms.

Stratification of the patients into LV systolic dysfunction and preserved cardiac function groups showed that the patients with LV systolic dysfunction had significantly lower HAP scores. Several studies have demonstrated that patients with LV systolic dysfunction exhibit functional impairment. Costa et al.^11^ demonstrated that patients with LV systolic dysfunction exhibit lower peak oxygen uptake and walk a shorter distance during the six-minute walk test. Another study^32^ indicated that CCC is associated with decreased physical activity levels. Notably, the HAP questionnaire in the present study showed satisfactory sensitivity in detecting functional changes associated with disease progression. In addition, the binary logistic regression analysis indicated that HAP score was the only functional variable that was associated with LV systolic dysfunction. These findings highlight the importance of HAP in clinical practice. 

The ROC curves plotted in the present study demonstrated that HAP shows acceptable accuracy in identifying LV systolic dysfunction in patients with CCC. A cut-off point of 56 points, which had a negative predictive value of 89.58%, was considered optimal for identifying such patients. This means that patients with CCC who present with HAP scores higher than 56 points have a 90% probability of not having LV systolic dysfunction. These results have important clinical implications for the management of Chagas disease because the HAP questionnaire can serve as a low-cost instrument for screening patients who require specialized cardiac evaluation. In contrast, owing to the low positive predictive value (24%) obtained in the analysis, values below the cut-off point are not appropriate for risk stratification because they may not accurately determine the severity of CCC. Nevertheless, we presented two other HAP score cut-off points for the identification of LV systolic dysfunction in patients with CCC: one with high sensitivity (70 points) and the other with high specificity (37 points). The establishment of these cut-off points can further assist in patient screening. For example, patient with a HAP score greater than 70 has a 92% probability of having preserved cardiac function. Despite these relevant findings, further studies are needed to validate the abovementioned cutoff points for patients with CCC.

A previous study^33^ has indicated that the Short-Form Health Survey shows good accuracy in identifying patients with LV systolic dysfunction. This demonstrates that patient-reported outcome measures, such as SF-36 and HAP scores, are valuable instruments in research and clinical practice and can be useful for monitoring disease progression.

The present study has some limitations. First, the study sample predominantly comprised patients with preserved functional performance. However, the study sample is representative of the population residing in an endemic area, where patients typically perform activities that require physical effort. Further research is needed to validate the established cutoff point and evaluate the use of self-report tools for identifying unfavorable outcomes. Moreover, future studies with different sample populations are needed to strengthen the validity of the evidence obtained in the present study. Second, the sample of the present study primarily consisted of elderly individuals; therefore, the generalizability of the study findings may be limited. Nevertheless, with the declining incidence of Chagas disease, aging populations in several regions, and advancements in patient management, it is anticipated that the population of patients with CCC will gradually become older. Notably, only a few previous studies were focused on older patients with CCC^12^. 

Despite these limitations, the present study is valuable because to our knowledge, it is the first study in which HAP was evaluated as a tool for identifying LV systolic dysfunction in patients with CCC, with the results demonstrating a significant difference in mean values between groups with severe clinical forms of the disease. It should be noted that Chagas disease is a neglected disease, and areas in which it is endemic are generally associated with low rates of human development, limited resources, and few technological devices. Therefore, low-cost tools are necessary for assessing functional performance in clinical setting in these areas. 

## CONCLUSION

HAP score is correlated with functional variables and can identify LV systolic dysfunction in patients with CCC. The optimal cutoff point for identifying patients with LV systolic dysfunction is 56 points. Patients with CCC who score higher than the cutoff point have a high probability (> 90%) of not having LV systolic dysfunction.
